# Empowerment or dependency? A systematic review of the impacts of intelligent assessment and generative AI on learners’ self-beliefs and cognitive agency in music education

**DOI:** 10.3389/fpsyg.2026.1776445

**Published:** 2026-02-17

**Authors:** Xiaoyu Peng, Kangrui Sun, Xin Shan, Junhan Zhang

**Affiliations:** 1 The Graduate School Arts and Culture, Sangmyung University, Seoul, Republic of Korea; 2 Department of Music, Sejong University, Seoul, Republic of Korea

**Keywords:** cognitive agency, cognitive offloading, dependence, educational technology, empowerment, music education, self-beliefs

## Abstract

Although artificial intelligence is fundamentally reshaping the ecology of music learning, existing research has disproportionately emphasized performance outcomes while underexamining psychological mechanisms, leaving the tension between technological empowerment and cognitive dependence theoretically underarticulated. Following PRISMA 2020, we systematically searched four databases and included 21 empirical studies to examine how three AI tool types—assessment-oriented AI, generative AI, and Comprehensive/adaptive AI—differentially shape learners’ self-beliefs and cognitive agency in music education. The evidence base remains geographically and developmentally concentrated: most studies were conducted in China and in higher education, while early childhood settings were absent. Using thematic analysis, we conducted cross-type comparisons and synthesized psychological pathways. Assessment-oriented AI most consistently strengthened ability beliefs via objectified, visualized feedback and positioned cognitive agency around self-monitoring, self-reactiveness, and self-reflectiveness. Generative AI tended to enhance value–attitude beliefs and intentionality by lowering technical barriers and reconfiguring learners’ creative roles toward aesthetic decision-making and output curation. Comprehensive/Adaptive AI more often supported forethought and sustained engagement by dynamically maintaining alignment between task challenge and learner capability. Across studies, psychological empowerment manifested as increased perceived competence and control, heightened motivation and engagement, and visible self-regulated learning behaviors. Cognitive dependence, however, emerged through outsourcing evaluative authority, score-driven goal distortion, algorithm-accommodating self-censorship, and attributional shifts that tether confidence to technological support. Developmental differences were also observed regarding dependence mechanisms: primary learners tended to perceive AI as a restrictive “scoring referee,” whereas higher education students demonstrated strategic agency in orchestrating AI assistance. Specifically, a critical construct–tool mismatch was identified: while assessment AI consistently supports self-reflectiveness, generative AI currently lacks sufficient evidence for fostering learners’ forethought. In light of the identified construct–tool mismatch, future research should prioritize addressing the paucity of evidence on how generative and adaptive AI foster forethought and intentionality, thereby clarifying whether such technologies ultimately reconstruct or erode learners’ cognitive agency.

## 1 Introduction

Across the long history of music education, from the apprenticeship model of oral transmission and embodied demonstration (Casas-Mas et al., 2022) to the collective instruction typical of modern conservatory-based systems (Utne-Reitan, 2022), each wave of technological mediation has reshaped the ecology of teaching and learning. Yet the introduction of AI into assessment and feedback workflows is again reconfiguring educational practice (Kamalov et al., 2023; Tay, 2024). This transformative potential has triggered a surge of international scholarly attention, as exemplified by the recent special issue on AI in music education (Bell and Richerme, 2025), which underscores the urgency of examining the philosophical and pedagogical implications of these technologies. Unlike conventional recording technologies or digitized scores, AI operates as an interactive and adaptive pedagogical agent, deeply intervening in instructional processes through real-time, multidimensional feedback on the assessment side (Pardue and McPherson, 2019) and creative collaboration on the generation side (Xu and Xu, 2024). In this context, AI not only provides immediate support as a form of cognitive scaffolding, effectively alleviating cognitive load during low-level skill practice, but also enables learners to allocate psychological resources to higher-order activities such as creative development and critical thinking (Merchán Sánchez-Jara et al., 2024). Meanwhile, AI-assisted tools have been associated with positive effects on learners’ learning motivation, self-regulation, and learning autonomy, suggesting a potential pathway of “psychological empowerment” (Ba et al., 2025; Yildiz Durak and Onan, 2025).

However, the same mechanisms may also carry risks of alienation: when learners over-rely on external algorithmic compensation for monitoring, judgment, and generative processes that should otherwise be carried out by the individual, technological support may shift from a developmental scaffold into a crutch that substitutes for agency, thereby inducing risks of agency degradation (Zhai et al., 2024). The resulting inflation of academic self-efficacy alongside a diminished sense of control—often accompanied by a higher tendency toward learned helplessness—may further erode the internal foundations of self-beliefs (Azeem and Abbas, 2025). More specifically, when AI-based digital evaluation is uncritically treated as an absolutely objective authority (Romeo and Conti, 2025), or when generative AI directly takes over core decision-making processes (Hou et al., 2025), this kind of substitutive use is more likely to consolidate into habitual dependence, impeding the internalization and transfer of cognitive capacities.

This duality of technological intervention touches on a central psychological paradox of music learning in the intelligent era: Is AI strengthening learners’ self-beliefs and cognitive agency, or is it evolving—through implicit substitution mechanisms—into a novel driver of dependence? Although prior studies have demonstrated that AI can significantly enhance the efficiency of skill acquisition and academic achievement (Dong et al., 2025; Zhou and Kim, 2024), two key limitations remain at the level of systematic synthesis. First, existing evidence integration exhibits a structural tendency to privilege outcomes over psychology, with studies often providing an overview based primarily on cognitive learning outcomes (Luo et al., 2025). Most reviews prioritize academic achievement or skill outputs as core endpoints (Chen and Cheung, 2025; Deng and Yu, 2023; Deng et al., 2025; Fleckenstein et al., 2023), yet offer insufficient mechanistic explanation of how AI may more deeply reshape learners’ psychological structures—such as the architecture of self-beliefs—thereby leaving the pathways of effects and their applicable boundary conditions unclear. Second, there is a lack of differentiated discussion across tool types. With the widespread adoption of generative AI, human–AI interaction has expanded from one-way error correction to complex co-creation; however, whether different AI tool types operate through systematically different mechanisms in shaping learners’ cognitive agency remains under-theorized and lacks a cross-type comparative and integrative framework.

Against this backdrop, the present study aims to move beyond a narrow validation of technological effectiveness to elucidate the key pedagogical and cognitive mechanisms that determine the direction of technological efficacy. Specifically, this study seeks to examine how different types of AI tools—namely assessment-oriented AI, generative AI, and comprehensive/adaptive AI—reshape learners’ self-beliefs and the boundaries of cognitive agency through specific interaction pathways, and to identify the mechanism chains under which empowerment reverses and becomes dependence. This effort is intended to bridge the gap between effects of technology application and psychological generative mechanisms, thereby providing a theoretical basis for ethics and instructional design in music education in the intelligent era. Accordingly, through a systematic review, this study addresses the following three core research questions (RQ):

*RQ1:* How do different types of AI tools in music education specifically shape learners’ self-beliefs and cognitive agency?

*RQ2:* What are the distinct behavioral and psychological manifestations associated with technological empowerment and cognitive dependence in music learning contexts?

*RQ3:* In music education, which human–AI interaction patterns and pedagogical contexts moderate the transition between empowering scaffolding and agency-eroding dependence?

## 2 Core conceptual definitions

The core conceptual definitions in this study are intended to provide a coherent theoretical coordinate system for subsequent evidence extraction and mechanism analysis, and to operationalize the psychological variables of self-beliefs and cognitive agency through a fine-grained dimensional specification. This approach is designed to prevent relevant discussions from remaining at a generalized, outcome-only description of “learning effects.”

### 2.1 Self-beliefs

In this study, self-beliefs are defined as a cognitive evaluative system in which individuals simultaneously appraise their own capability level and the value of the task. Within this system, ability beliefs constitute a foundational predictor of individual action. Anchored primarily in Bandura’s (1977) social cognitive theory, this study treats self-efficacy as the core representation of ability beliefs, focusing on learners’ efficacy expectations and sense of control regarding whether they can successfully complete specific tasks when confronted with technical challenges in music learning. It reflects learners’ confidence in their current skill level and in their capacity to overcome difficulties. However, a complete system of self-beliefs is not confined to capability judgments about “whether I can do it,” but must also incorporate value appraisals concerning “whether I want to do it” (Wigfield and Eccles, 2000). According to Eccles and Wigfield’s expectancy–value theory, the value dimension functions as a central driver of the motivational system. This dimension encompasses intrinsic interest, the utility of the task for future goals, and perceived costs; fundamentally, it represents a dynamic process through which individuals align external tasks with internal “self-schemata”—that is, evaluating whether engaging in the activity is congruent with one’s identity and long-term goals (Eccles, 2009). Accordingly, ability beliefs and value-attitude beliefs do not operate in isolation; rather, they work synergistically to shape learners’ persistence and engagement in technology-mediated learning contexts (Wigfield and Eccles, 2000).

### 2.2 Cognitive agency

In this study, cognitive agency is defined as learners’ autonomous psychological capacity to regulate learning processes and reflect on outcomes within music learning contexts, emphasizing learners’ self-directed and self-regulatory functioning as “actors” rather than passive recipients. Drawing on Bandura’s (2001) social cognitive account of human agency, this study operationalizes cognitive agency into four core features. Intentionality and forethought represent the point of action initiation and a planning orientation, capturing learners’ expectations regarding goal setting, strategy selection, and future outcomes (e.g., actively deciding to interpret a piece with a specific emotional tone and planning phrasing strategies before playing). Self-reactiveness and self-reflectiveness correspond to self-monitoring, regulation, and metacognitive evaluation during task execution, capturing learners’ process control while acting (e.g., adjusting bow pressure in real-time when hearing a scratchy sound), as well as their reflective judgments after action regarding outcomes, causes, and directions for improvement (e.g., analyzing why a specific rhythm was rushed and formulating a correction plan). This four-dimensional structure serves as the micro-analytic framework of the present study to examine, in detail, learners’ internal psychological processing during interactions with AI tools—from action initiation and goal pre-specification to process regulation and outcome evaluation—thereby enabling a more precise characterization of the full profile of cognitive agency and its dynamic changes under technological intervention (Bandura, 2001).

## 3 Theoretical framework

**Figure 1:**
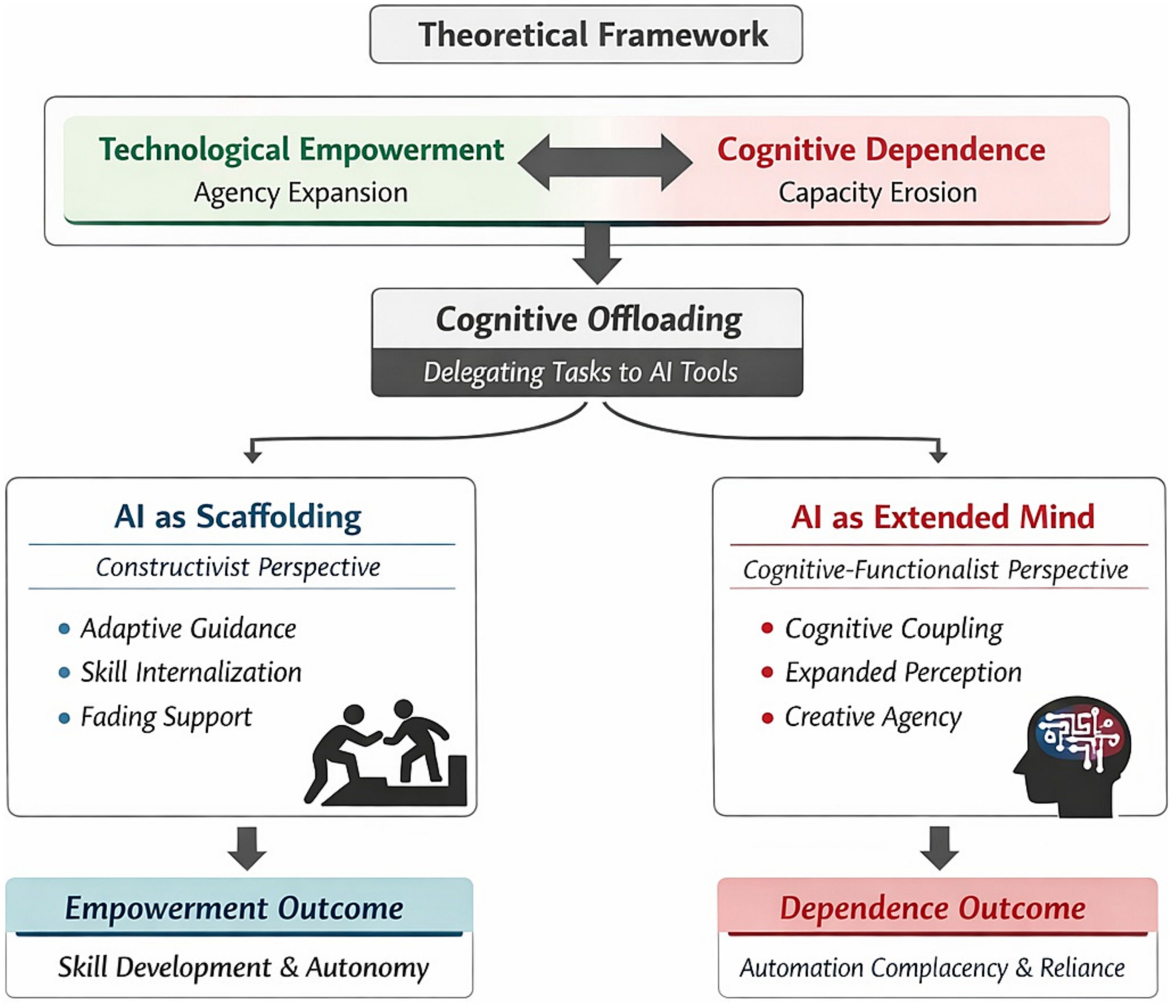
Integrated theoretical framework of empowerment and dependence in AI-supported music learning.

To elucidate the complex tension between technological empowerment and cognitive dependence in music learning, this study integrates three complementary theoretical perspectives: cognitive offloading, scaffolding, and the extended mind (see Figure 1). We posit cognitive offloading as the fundamental mechanism of interaction, wherein learners transfer processing demands to AI tools. The trajectory of this offloading—whether it leads to empowerment or dependence—is subsequently interpreted through the lenses of scaffolding and the extended mind. Specifically, we examine whether offloading functions as a pedagogical scaffold that supports the internalization of skills and eventually fades (fostering empowerment), or conversely, acts as a permanent substitute that creates reliance. The extended mind perspective further frames the ideal outcome of this interaction, describing a state where AI serves as a stable cognitive partner that expands, rather than replaces, the learner’s aesthetic agency.

### 3.1 Cognitive offloading: the trade-off between efficiency gains and capability loss

Cognitive offloading refers to the process by which individuals reduce cognitive demands by using physical actions or external environments (e.g., tools and technologies) to alter information-processing requirements (Risko and Gilbert, 2016). In music learning, AI-driven automated feedback (e.g., pitch-visualization software and intelligent accompaniment systems) functions as an external cognitive resource, allowing learners to offload low-level monitoring tasks (e.g., Is my intonation correct?) to algorithms. From the perspective of cognitive load theory, integrated presentation and adaptive guidance can reduce extraneous load caused by split attention, thereby freeing limited working memory resources for higher-order musical expression and interpretation (Sweller, 2011).

On the other hand, the cognitive miser assumption suggests that humans tend to minimize cognitive effort whenever possible. When music learners rely excessively on automated feedback to substitute for internal auditory monitoring, they may fall into the trap of automation complacency—that is, overtrusting the accuracy of automated systems, which in turn reduces vigilance in monitoring (Parasuraman and Manzey, 2010).

Accordingly, within the present framework, we define dependence as the state in which cognitive offloading impedes the internalization of learners’ core musical cognitive capacities (e.g., audiation and self-correction abilities). This directly threatens learners’ cognitive agency, shifting them from being the “director” of musical action to becoming an “executor” of the system.

### 3.2 Mechanisms of empowerment: AI as scaffolding and extended mind

To explain how AI can promote rather than diminish agency, this study draws on complementary perspectives from constructivism and cognitive science.

First, the concept of scaffolding originates in the classic definition by Wood et al. (1976): in the process of acquiring new skills, an instructor (or the environment) provides temporary support—through segmented guidance, error marking, and task decomposition—to carry component skills that learners cannot yet perform independently, and gradually withdraws such support as competence increases (Wood et al., 1976). This definition is not limited to human–human interaction. In technology-enhanced learning environments, automated assessment and adaptive pathways can function as technology-based scaffolding by providing conceptual, procedural, and metacognitive support in ways that are contextualized and fade over time (Sharma and Hannafin, 2007). In music education, AI is not merely a feedback tool; grounded in the notion of the zone of proximal development (ZPD), it can support learners in completing tasks at a level beyond their current independent capability (Vygotsky, 1978). Moreover, research on scaffolding for self-regulated learning and metacognition indicates that computerized diagnostic–adaptive–fading support helps cultivate key capacities such as goal setting, monitoring, and reflection (Azevedo and Hadwin, 2005).

Complementing this account, the “extended mind” and “distributed cognition” perspectives provide an ontological explanation for AI-enabled empowerment. Clark and Chalmers (1998) proposed the extended mind thesis, arguing that under stable and reliable coupling, external tools can become constitutive components of cognitive processes. In the context of educational technology, Salomon et al. (1991) further distinguished between immediate performance gains achieved through collaboration with technology and longer-term changes that can be transferred and internalized as individual competence. From this perspective, AI tools are not merely external aids; under conditions of stable coupling and high interdependence, they can form an intelligent partnership with the learner.

It is important to note that this study adopts a cognitive-functionalist view of the extended mind rather than a post-humanist perspective. We posit AI as a tool that extends human processing capacity under human control, without attributing autonomous intentionality or moral agency to the technology itself.

Therefore, when music learners establish a constructive interactive relationship with AI systems—namely, when learners actively use AI-generated analyses to reflect on their own performance rather than passively following instructions—AI becomes an extension of learners’ cognition. Such cognitive coupling expands learners’ perceptual and evaluative capacities, enabling them to detect subtle temporal deviations or timbral changes. Within this framework, empowerment is reconceptualized as follows: through human–AI collaboration, learners’ cognitive boundaries are extended, thereby strengthening their self-awareness and creativity as aesthetic agents.

## 4 Method

This study was conducted and reported in strict accordance with the Preferred Reporting Items for Systematic Reviews and Meta-Analyses (PRISMA 2020) statement (Page et al., 2021).

### 4.1 Information sources and search strategy

A systematic literature search was completed in early December 2025. The search covered four core academic databases: Web of Science (Core Collection), APA PsycINFO, ERIC, and Scopus.

The search-term construction followed the PICOS framework and operationalized the key concepts into four logical blocks: (1) Context, specifying the disciplinary domain of music; (2) Participants, focusing on learners across educational stages; (3) Intervention, encompassing a broad range of AI technologies; and (4) Outcomes, capturing multidimensional representations of self-beliefs and cognitive agency.

Drawing on controlled vocabularies and commonly used terms in prior research, the search strategy expanded synonyms, abbreviations, and representative tool names (e.g., GenAI, ChatGPT, Suno). Pilot searches were iteratively conducted to calibrate recall and precision, resulting in a cross-database search string. The final query was: (Music* OR “musical instrument” OR singing OR vocal OR piano OR compose*) AND (student* OR learner* OR pupil* OR undergraduate OR postgraduate) AND (“artificial intelligence” OR AI OR “generative AI” OR GenAI OR Suno OR ChatGPT OR “intelligent tutor*” OR “intelligent tutoring system*” OR “automated feedback” OR “automatic* assessment” OR “intelligent tutoring” OR chatbot* OR “large language model*” OR “AI feedback” OR “computer-assisted instruction”) AND (“Self-belief*” OR “belief*” OR “self-efficacy” OR “self-regulated learning” OR “confidence” OR “competence” OR “self-concept” OR “self-esteem” OR “mindset” OR “motivation” OR “Agency” OR “epistemic agency” OR “autonomy” OR “ownership” OR “authorship” OR “self-determination” OR “independence” OR “control” OR “Decision-making” OR “Critical thinking” OR Validation OR Reflection OR “dependency” OR “reliance” OR “experience” OR “perception” OR “attitude” OR “satisfaction” OR “engagement” OR “perspective” OR “viewpoint”).

### 4.2 Study selection and quality control

To ensure objectivity and consistency in the study-selection process, two reviewers independently screened the records following a two-stage procedure: an initial screening of titles/abstracts and a subsequent full-text screening. Inter-rater agreement was assessed using Cohen’s kappa, indicating good consistency (*κ* = 0.86). Any discrepancies were resolved through adjudication by a third-party expert to reach a final decision.

The first stage (title/abstract screening) evaluated whether studies met the basic inclusion criteria: (a) whether the research context fell within music education or music learning; (b) whether participants were students/learners; (c) whether an AI-related intervention was present; and (d) whether the study included psychological indicators, design elements, or plausible discussion cues relevant to self-beliefs or cognitive agency. This stage was intended to rapidly exclude clearly out-of-scope records while retaining potentially relevant studies for full-text assessment.

**Figure 2:**
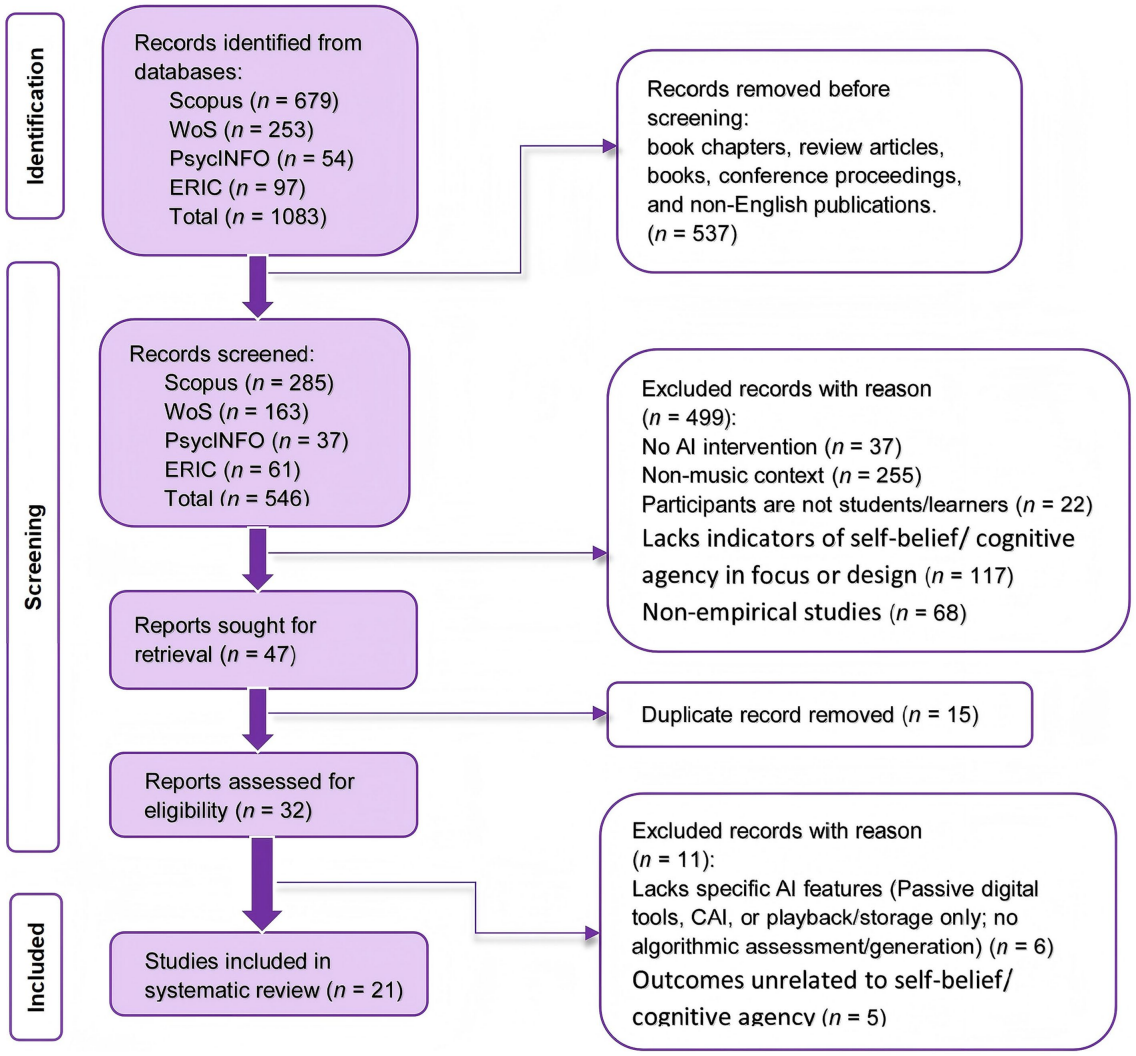
PRISMA diagram.

The second stage (full-text screening) applied a more stringent determination based on the present review’s core definition of AI tools. To delineate the boundary between empowerment and dependence, this review restricted AI tools to technological forms characterized by algorithmic intervention and autonomous feedback—namely, tools that function not merely as information carriers but that can simulate human auditory perception or logical judgment via algorithms, thereby directly intervening in and partially assuming learners’ cognitive processing (i.e., involving substantial cognitive offloading). Under this restriction, discussions of dependence leading to a loss of agency become epistemologically meaningful. Accordingly, the review strictly distinguished between technology as a carrier (e.g., tools limited to resource presentation, audio playback, data storage, social sharing, or peer-assessment platforms primarily driven by human feedback) and technology as a cognitive agent. For example, the seemingly relevant study *Application of Artificial Intelligence and Wireless Networks to Music Teaching* contained AI in the title, yet its abstract and full text primarily described the convenience of resource access in a smart classroom” environment (tablets, wireless networks, and information retrieval) rather than algorithmic assessment or generative intervention in learning; it was therefore excluded at the full-text stage. Similarly, applications that provide only audio playback, storage, or social sharing functions (e.g., mobile social platforms for peer feedback) were classified as conventional digital learning tools, whose feedback mechanisms rely mainly on teachers or peers rather than algorithms and do not entail substantial cognitive offloading; thus, they were not included in this review. Detailed reasons and counts for exclusions at each stage are presented in Figure 2.

### 4.3 Data extraction and analytic approach

A standardized data-extraction form was used to code all studies included in the final sample. Extracted fields comprised: (1) bibliographic characteristics (title, authors, publication year, and country/region); (2) methodological characteristics (sample size, gender composition, educational stage, and study design: qualitative, quantitative, or mixed methods); (3) intervention characteristics (the specific AI tool type used and its functional positioning); (4) focal variables (the specific dimensions of self-beliefs and/or cognitive agency addressed); and (5) study findings (empirical results pertaining to technological empowerment and/or technological dependence).

Data synthesis employed thematic analysis. Through inductive coding and thematic consolidation of extracted qualitative and quantitative findings, the analysis aimed to reconstruct the psychological landscape of music learning under AI intervention along two overarching dimensions: technological empowerment and technological dependence.

## 5 Results

### 5.1 Descriptive results

**Figure 3:**
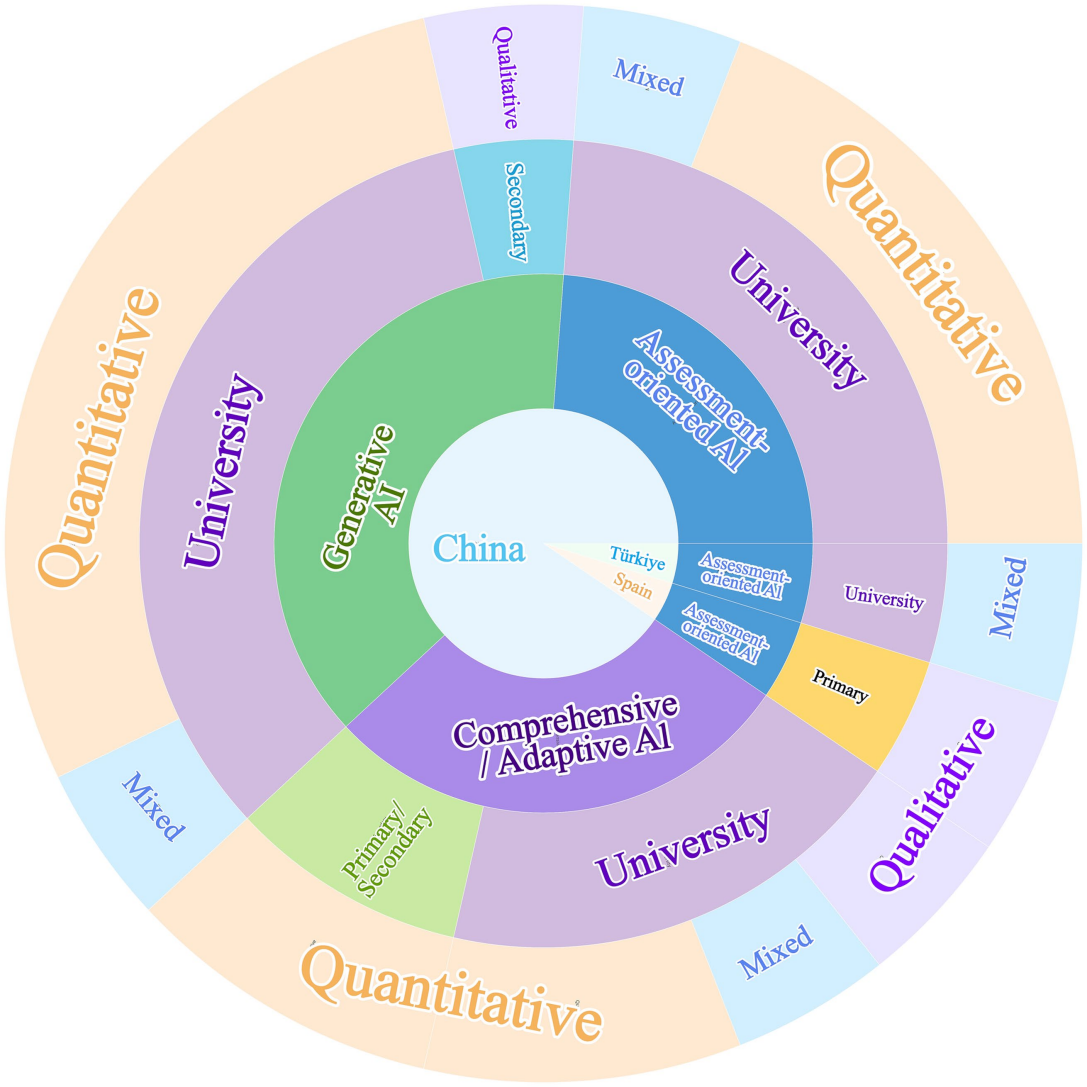
Study characteristics across contexts and designs.

Across the 21 included studies, the geographical distribution was highly concentrated. The vast majority of studies were conducted in China (*n* = 19), with only a small number from Türkiye (*n* = 1) and Spain (*n* = 1) (see Table 1 and Figure 3). Participants were predominantly drawn from higher education contexts (university level, *n* = 17), suggesting that empirical evidence on this topic has primarily accumulated in university classrooms, professional music training settings, or university students’ self-directed learning contexts. By comparison, only one study focused on secondary education and one on primary education, while two studies covered mixed primary/secondary cohorts, indicating that evidence from basic education remains relatively limited. This geographical concentration mirrors the rapid integration of Ed-Tech in East Asian exam-oriented systems, where quantitative scoring is highly valued. Similarly, the predominance of higher education samples reflects the accessibility of university participants and their higher degree of self-directed learning time compared to K-12 students. Sample sizes varied widely, ranging from case studies (*n* = 4) to large-scale questionnaire surveys (*n* = 1,125), and the overall proportion of female participants was higher than that of male participants. This gender distribution reflects typical music education demographics and may influence findings regarding receptivity to affective AI features, as female learners have been observed to exhibit distinct motivational responses to emotional visualization tools (Liu, 2025).

**Table 1:** Characteristics of included studies.

No.	Title	Authors (Year)	Country	Education stage	Design	Purpose	AI tool category
S1	AI-assisted feedback and reflection in vocal music training: effects on metacognition and singing performance	Li et al. (2025)	China	University	Mixed	Examine effects of LLM-based dialogic feedback plus an audio comparison tool on metacognition and singing performance in vocal training	Comprehensive/Adaptive AI
S2	Algorithmic Foucault: Digital Feminism, the Panopticon, & the Role of AI in Shaping Musical Identities & Pedagogies	Zhu (2025)	China	Secondary	Qualitative	Explore generative AI as both a creative tool and an algorithmic monitoring mechanism shaping students’ musical identities and pedagogy	Generative AI
S3	Automatic generation of music education content based on deep learning algorithms	Yu (2025)	China	Primary/Secondary	Quantitative	Evaluate the quality and personalization of a deep-learning-based music education support system	Comprehensive/Adaptive AI
S4	Artificial intelligence and creativity: piano teaching with augmented reality applications	Cui (2023)	China	University	Quantitative	Assess AR-based piano learning apps for engagement and perceived skill gains	Assessment-oriented AI
S5	Does AI-assisted creation of polyphonic music increase academic motivation? The DeepBach graphical model and its use in music education	Yuan (2024)	China	University	Quantitative	Test whether DeepBach-assisted polyphonic composition increases academic motivation and supports learning	Generative AI
S6	Enhancing musical creativity through AI: a chain-mediation model of self-efficacy and emotional intelligence	Wang and Zhang (2025)	China	University	Quantitative	Model pathways from AI use to musical creativity via self-efficacy and emotional intelligence	Generative AI
S7	Exploration of the multiple integration mode of modern intellectualised music teaching and traditional music culture	Zhang (2024)	China	University	Quantitative	Build and evaluate an intelligent framework integrating traditional music culture and improving interest/efficiency	Comprehensive/Adaptive AI
S8	Exploring the impact of AI-assisted practice applications on music learners’ performance, self-efficacy, and self-regulated learning	Ou et al. (2025)	China	University	Mixed	Examine AI practice app impacts on performance, self-efficacy, and self-regulated learning	Assessment-oriented AI
S9	How anthropomorphic AI features affect music students’ acceptance: a study among Chinese undergraduates	Tan et al. (2025)	China	University	Mixed	Investigate how anthropomorphic features shape AIGC acceptance and uncanny valley effects	Generative AI
S10	Hybrid models of piano instruction: How combining traditional teaching methods with personalized AI feedback affects learners’ skill acquisition, self-efficacy, and academic locus of control	Wang S. (2025)	China	University	Quantitative	Test hybrid instruction + AI feedback on skill acquisition, self-efficacy, and locus of control	Assessment-oriented AI
S11	Integration of AI GPTs in music education and their impact on students’ perception and creativity	Wang X. (2025)	China	University	Quantitative	Evaluate integrating ChatGPT into teaching on creativity and technology perceptions	Generative AI
S12	Modern AI program Chinese choral arts: cognitive training and motivation of college choristers	Liu (2025)	China	University	Quantitative	Assess AI-supported choral training effects on cognitive training and motivation	Generative AI
S13	Musical education and academic motivation in highly developed AI technology	Zhang and Li (2025)	China	University	Quantitative	Evaluate Flowkey effects on piano-learning motivation	Assessment-oriented AI
S14	Psychological factors influencing successful music learning using deep learning technologies	Xu and Xu (2024)	China	University	Quantitative	Compare deep-learning tools vs. traditional methods on academic self-efficacy, wellbeing, and achievement in composition tasks	Generative AI
S15	Research on Human-centered Design in College Music Education to Improve Student Experience of Artificial Intelligence-based Information Systems	Qian (2023)	China	University	Qualitative	Identify integration effects, challenges, and human-centered design principles for AI-based information systems in music education	Comprehensive/Adaptive AI
S16	Seeing Sounds: The Effect of Computer-Based Visual Feedback on Intonation in Violin Education	Aksoy (2023)	Türkiye	University	Mixed	Test computer-based visual feedback effects on violin intonation and learners’ experiences	Assessment-oriented AI
S17	Self-regulation strategies and behaviors in the initial learning of the viola and violin with the support of software for real-time instrumental intonation assessment	López-Calatayud and Tejada (2024)	Spain	Primary	Qualitative	Examine SRL strategies in beginner instrumental learning supported by real-time intonation assessment software	Assessment-oriented AI
S18	Sound, Screen, Support: Smart Studio Environments and AI Integration in Motivating Chinese Piano Students	Zhang (2025)	China	Primary/Secondary	Quantitative	Test smart studio environments and AI integration on motivation/engagement via confidence and career utility	Comprehensive/Adaptive AI
S19	The Role of AI and Voice-Activated Technology in Religious Education in China: Capturing Emotional Depth for Deeper Learning	Wang and Kang (2025)	China	University	Quantitative	Explore AI/voice analytics for emotional depth capture and effects on affect and performance	Assessment-oriented AI
S20	The Role of Technophilia, Positive Experience and Flow in Enhancing Learning Efficiency in AI-Based Music Education: A Structural Equation Approach	Wang and Liu (2025)	China	University	Quantitative	SDT-based model linking technophilia/experience → flow → learning efficiency in AI-based music education	Generative AI
S21	Unlocking the Beat: How AI Tools Drive Music Students’ Motivation, Engagement, Creativity and Learning Success	Chen (2025)	China	University	Quantitative	Examine how AI tools relate to motivation, engagement, creativity, and learning success	Comprehensive/Adaptive AI

In terms of research design, quantitative studies were predominant (*n* = 14), most commonly employing experimental/quasi-experimental designs or cross-sectional surveys. Mixed-methods studies (*n* = 4) combined quantitative evidence with qualitative materials (e.g., interviews and learning logs) to provide more in-depth interpretation, whereas qualitative studies (*n* = 3) primarily used case study approaches, semi-structured interviews, and thematic analysis to examine learners’ subjective experiences.

The AI tools examined were diverse and can be grouped into three major categories. First, assessment-oriented AI (*n* = 7), represented by tools such as Violy, Flowkey, Plectrus, and pitch-visualization software, primarily provided real-time pitch/rhythm analysis and visualized feedback. Second, generative AI (*n* = 8), represented by DeepBach, MusicVAE, Suno, and various AIGC platforms, was mainly used for automatic generation of melodies, harmonies, and accompaniments to support creative work. Third, comprehensive/adaptive AI systems (*n* = 6) integrated functions such as adaptive pathway recommendation and/or intelligent dialogue.

### 5.2 Differential effects of AI tool types on self-beliefs and cognitive agency

#### 5.2.1 Assessment-oriented AI

For assessment-oriented AI, the core mechanism driving psychological change is objectified feedback. By converting abstract auditory signals into concrete visual data or scores, these tools primarily reshaped learners’ ability beliefs and their self-reactiveness/self-monitoring.

First, the central psychological effect of assessment-oriented AI derives from a feedback structure that is visible, quantifiable, and comparable. It transforms learning processes that traditionally rely heavily on subjective listening or teachers’ verbal judgments into external evidence that learners can repeatedly verify, thereby directly strengthening ability beliefs. At the level of self-beliefs, assessment-oriented AI markedly enhanced learners’ perceived competence and sense of control. Studies reported that by visualizing otherwise invisible intonation or rhythmic deviations, AI functioned as an externalized superego, reducing uncertainty associated with reliance on subjective feelings during practice (S16, S19), thereby lowering anxiety and increasing calmness (S19). This what-you-see-is-what-you-get feedback mechanism substantially reduced the cognitive threshold, enabling beginners with no prior experience to confirm mastery of specific skills (e.g., 90% of students reported that they had mastered technical skills), thus supporting robust self-efficacy (S4). Similarly, a violin practice app that provided real-time intonation/rhythm analysis and comprehensive progress reports significantly increased learning/performance self-efficacy: learners could see themselves improving each day, and such stable evidence of progress not only increased confidence but also made ability beliefs easier to sustain (S8). In blended learning contexts, Yousician’s personalized feedback likewise significantly improved self-efficacy (S10). Moreover, a Flowkey study grounded in the MUSIC model suggested that assessment-oriented feedback could also strengthen learners’ perceptions of process control and success expectancy, thereby elevating both ability beliefs and value-attitude beliefs (S13).

At the level of cognitive agency, the shaping effects of assessment-oriented AI were typically concentrated in self-reactiveness and self-reflectiveness, and in some studies extended to forethought. Assessment-oriented AI could, to some extent, support a closed-loop cycle of self-monitoring → immediate adjustment → post-hoc review. Interview evidence in S8 indicated that learners used app-generated data to set more specific goals, engaged in immediate error correction during performance, and conducted self-evaluation and reflection after practice based on scores and reports, thereby forming a relatively complete self-regulated learning cycle. For younger beginners, real-time scoring software also translated practice into trackable goals, fostering goal orientation and execution regulation (S17). When students used scores or waveform visualizations to calibrate their performance, they were effectively engaging in deep metacognitive monitoring (S16). Additionally, the perceived sense of control over the learning process directly promoted learners’ willingness to engage in independent after-class practice (S13, S16).

#### 5.2.2 Generative AI

Unlike assessment-oriented tools, the influence of generative AI more closely resembled a reconfiguration of what kind of musical activity learners are engaged in, and in what role. Accordingly, generative AI primarily reshaped learners’ value-attitude beliefs and intentionality.

At the level of self-beliefs, generative AI lowered technical barriers and thereby enhanced learners’ academic motivation and technology affinity. For students lacking advanced compositional expertise, AI served as a creative prosthesis enabling them to overcome technical obstacles and attain successful creative experiences (S2). A prototypical example is DeepBach-supported polyphonic composition: when the system assumed substantial rule-based computation, students shifted from passively executing rules to actively making aesthetic choices, with a significant increase in academic motivation (S5). This reflects a pathway through role transformation that strengthens both value-attitude beliefs and intentionality. Chain-mediation findings further indicated that generative AI created successful experiences through immediate feedback and task decomposition, first enhancing music self-efficacy and then facilitating creativity via psychological capacities such as emotional intelligence (S6, S14). Notably, when students exhibited higher technology affinity, AI environments could effectively elicit flow experiences; such intensely positive experiences strongly reinforced intrinsic value-attitude beliefs toward learning (S20).

At the level of cognitive agency, generative AI liberated learners from laborious rule computation or basic material generation, enabling them to focus on higher-order intentionality—namely, aesthetic decision-making and knowledge restructuring. Studies found that learners shifted from rule executors to subjects of aesthetic decision-making, actively exploring and reorganizing AI-generated materials (S5). This release of agency was associated with stronger proactivity in practice and creative work (S12). At the same time, through self-directed prompting and reflection, learners’ capacity to reorganize information as cognitive agents was further strengthened (S11).

Any alternative text (alt text) provided alongside figures in this article has been generated by Frontiers with the support of artificial intelligence and reasonable efforts have been made to ensure accuracy, including review by the authors wherever possible. If you identify any issues, please contact us.

#### 5.2.3 Comprehensive/adaptive AI

Comprehensive/adaptive AI systems (e.g., intelligent recommendation algorithms and systems integrating SRL support) operated through mechanisms of difficulty regulation and personalized pathways. While sustaining learning motivation, these systems primarily strengthened learners’ forethought and active exploration.

At the level of self-beliefs, such tools maintained tasks within a productive challenge zone by precisely matching learners’ capability levels, effectively reducing frustration and avoidance behaviors (S3). This adaptive support not only improved immediate learning attitudes but also strengthened learners’ perceptions of the career utility of the technology—namely, learners not only believed they could learn well but also believed what they were learning had long-term professional value (S18). These positive perceptions directly predicted higher levels of learning engagement (S21).

At the level of cognitive agency, comprehensive/adaptive AI enhanced learners’ forethought and intentionality by reducing inefficient searching and providing personalized pathways. Evidence indicated that intelligent recommendation substantially increased the proportion of time devoted to self-directed learning and stimulated learners’ willingness to explore proactively (S7). When systems were designed to support goal setting and pathway planning, they helped learners develop a clear sense of learning goals (S15). Importantly, when such technologies were combined with instructional interventions such as reflective journals, external algorithmic recommendations were more likely to be internalized into learners’ self-directed strategy optimization, thereby closing the loop from technological assistance to cognitive independence (S1).

### 5.3 Manifestations of psychological empowerment and cognitive dependence

Across the included studies, psychological empowerment most commonly manifested as three observable categories of learner change. The first concerns perceived gains in capability and control: learners became more confident that they could complete tasks and maintain control over the process, for example by using immediate error correction or visualized evidence to consolidate perceived competence and enhance self-efficacy (S4, S8, S10, S14) (see Table 2). In some contexts, this also appeared as a stronger sense of control accompanied by reduced anxiety and increased calmness (S19), or broader improvements in psychological wellbeing (S14). The second category involves positive shifts in value and attitudes: increases in motivation, interest, positive attitudes, intention to continue use, participation, and engagement (S3, S5, S13, S15, S16, S18, S20, S21). This included attitude shifts driven by a better-calibrated level of challenge (“the challenge feels more appropriate, so I am more willing to practice”; S3), increases in value judgments that tools are more useful, more enjoyable, and more supportive of learning (S13, S18), and high-involvement states emerging under conditions of technology affinity and positive experiences (S20). The third category is the outward expression of agentic learning behaviors: learners practiced more proactively, showed greater willingness to complete tasks independently, were more likely to continue practicing outside class, and, in creative contexts, exhibited greater willingness to experiment and express themselves (S4, S5, S6, S7, S11, S12, S16, S21). Examples included shifts from executor roles toward more active creative participation and knowledge application (S5, S11, S21), or marked increases in self-directed learning time facilitated by recommendation systems (S7).

**Table 2:** Evidence mapping of self-beliefs, cognitive agency, and empowerment/dependence manifestations.

AI tool category	Study index	Self-belief dimension	Cognitive agency dimension	Basis	Empowerment/Dependence (refined manifestations)
Assessment-oriented AI	4	Ability beliefs	Self-reflectiveness	Belief basis: Competence confirmation (90% of students confirmed they mastered specific skills). Agency evidence: Independent self-learning and self-evaluation (score reading and problem solving rely on self-evaluation).	Empowerment: AI reduces time/space constraints and turns abstract theory into concrete audio-visual feedback, lowering cognitive load and enabling beginners’ achievement and self-directed learning.
	8	Ability beliefs	Forethought; Self-reactiveness; Self-reflectiveness	Belief basis: Performance/learning self-efficacy (MPSE/MLSE): explicit efficacy-scale measurement. Agency evidence: Goal setting (data feedback helps set more specific, quantifiable practice goals); real-time monitoring (immediate error correction reduces reliance on delayed feedback); objective evaluation (AI scoring as an “objective mirror” reduces self-assessment bias).	Empowerment: AI supports the metacognitive cycle and autonomous practice; objective scoring provides quantifiable progress evidence, strengthening performance confidence and self-regulation.
	10	Ability beliefs	—	Belief basis: Self-efficacy and Academic Locus of Control.	Mixed (Empowerment + Dependence): Empowerment via detailed, immediate, personalized feedback that corrects errors and builds mastery experiences; Dependence risk: learners may become reliant on AI validation/feedback for confidence and task completion.
	13	Ability beliefs; Value–attitude beliefs	Intentionality	Belief basis: Empowerment/control and success expectation (ability dimension in the MUSIC model); interest and usefulness (value dimension in the MUSIC model). Agency evidence: Empowerment/control (having “control” over the learning process).	Empowerment: Strengthens perceived control over the learning process, which directly enhances intrinsic motivation and proactive engagement.
	16	Value–attitude beliefs	Self-reflectiveness	Belief basis: Motivation and willingness to use. Agency evidence: Visualization-based self-diagnosis (turning auditory problems into concrete targets provides opportunities for self-evaluation).	Empowerment: Visualizes “abstract intonation” to enable monitoring and self-evaluation; learners report motivational benefits, time-efficiency gains, and intention to continue independent use.
	17	—	Forethought; Self-reactiveness; Self-reflectiveness	Agency evidence: Forethought phase (planning the day’s focus before practice; SRL coding); execution control (using scores as an external regulator to fine-tune pitch during practice); self-judgment (attribution analysis based on scores to evaluate effectiveness).	Mixed (Empowerment + Dependence): Empowerment as software acts as an “external regulator,” turning practice into visible goals and enabling self-diagnosis and strategy adjustment; Dependence/limitation: “production deficit” (ignoring feedback) shows effectiveness depends on sustained learner attention and active uptake.
	19	Ability beliefs	Self-reactiveness	Belief basis: Sense of control and calmness (reduced anxiety as a physiological indicator of high control). Agency evidence: Emotion regulation (objective certainty helps reduce anxiety and maintain calmness).	Empowerment: Objective feedback reduces uncertainty, improves skills and mental state (calmness/confidence), strengthening persistence during practice.
Generative AI	2	Ability beliefs	—	Belief basis: Confidence (AI provides an extended sense of confidence in expression and creative capability).	Mixed (Empowerment + Dependence): Empowerment by lowering creative barriers—AI functions as a “creative prosthesis” that amplifies expressive drive and emotional resonance; Dependence risk: bias exposure and “algorithm-pleasing” behavior (Panopticon metaphor) may push internalization of platform rules and conformity, undermining agency.
	5	Value–attitude beliefs	Intentionality	Belief basis: Academic motivation (based on the ARCS model, emphasizing attention and relevance). Agency evidence: Aesthetic decision-making (AI handles technical details; students become the agents of aesthetic decisions).	Empowerment: AI simplifies complex polyphonic creation, freeing students from rule-calculation to focus on aesthetic choices; lowered threshold boosts confidence and interest.
	6	Ability beliefs	Intentionality	Belief basis: Music self-efficacy (directly measured as the core mediator variable). Agency evidence: Higher-level decision-making (AI as scaffolding; students focus on higher-level musical decisions).	Empowerment: Reduces technical complexity and provides immediate feedback, accumulating mastery experiences that raise self-efficacy and encourage innovative experimentation.
	9	Value–attitude beliefs	Self-reactiveness	Belief basis: Acceptance and the Uncanny Valley effect (affective attitudes toward technology and psychological cost). Agency evidence: Hybrid strategy regulation (using mixed human–AI review to cope with “uncanny valley/algorithm limitations”).	Mixed (Empowerment + Dependence): Empowerment through personalization and emotional stimulation that increases interactivity and motivation; Dependence/psychological challenge: learners may seek AI approval, while uncanny-valley discomfort creates a barrier that shapes usage and regulation.
	11	—	Self-reflectiveness	Agency evidence: Interactive reflection (reviewing ChatGPT-generated content prompts self-reflection).	Empowerment: High-quality feedback helps learners identify errors promptly and focus on problem areas, reducing teacher dependence and improving autonomous learning efficiency; also highlights need for Q&A tuning and user training.
	12	—	Intentionality	Agency evidence: “Motivation” and “decision rights in improvisation.”	Mixed (Empowerment + Dependence): Empowerment via auto-generation/adaptive feedback that lowers training thresholds and increases engagement; Dependence risk: concern about AI replacing human wisdom and “template-like” outcomes if overrelying on generated accompaniment.
	14	Ability beliefs	—	Belief basis: Academic self-efficacy (directly measuring confidence in completing tasks).	Empowerment: Deep-learning tools break creative monotony and scaffold early-stage technical barriers, improving confidence in complex tasks and enhancing wellbeing.
	20	Value–attitude beliefs	Self-reactiveness	Belief basis: Technophilia, flow, and positive experience (extreme intrinsic value and attitudinal experience). Agency evidence: Flow (high-intensity attentional control and sustained engagement).	Empowerment: AI as a supportive environment satisfies SDT-related psychological needs and promotes intrinsically driven flow, increasing efficiency and immersion.
Comprehensive/Adaptive AI	1	—	Intentionality; Self-reactiveness; Self-reflectiveness	Agency evidence: Strategy optimization (students actively use feedback for strategy optimization); self-monitoring (real-time self-monitoring in singing training); metacognition enhancement (reflection logs and waveform comparison improve metacognition).	Empowerment: AI scaffolds learning through dialogic feedback (e.g., breathing), prompting self-monitoring and strategy optimization; assessment skills are internalized, strengthening agency.
	3	Value–attitude beliefs	Intentionality	Belief basis: Learning motivation and attitude. Agency evidence: Voluntary extension (reduced avoidance and voluntary extension of practice time).	Empowerment: Adaptive paths provide appropriately challenging tasks (ZPD), reducing frustration and strengthening confidence and sustained learning motivation.
	7	—	Intentionality	Agency evidence: Active exploration (self-study time increased from 51 to 77%).	Empowerment: Precise recommendations reduce ineffective search, shifting time from entertainment to self-study and strengthening exploratory initiative.
	15	Value–attitude beliefs	Forethought	Belief basis: Enthusiasm and motivation (intrinsic value reported in qualitative interviews). Agency evidence: Clear learning path (personalized recommendations help set clear goals and take responsibility for the learning trajectory).	Mixed (Empowerment + Dependence): Empowerment via personalization and real-time feedback that strengthens initiative and goal management; Dependence/risks: concerns about teacher replacement, ethics, and algorithmic fairness.
	18	Ability beliefs; Value–attitude beliefs	Forethought	Belief basis: Confidence (as a core mediator variable); career utility (explicit instrumental-value measurement). Agency evidence: Goal engagement (future-oriented, goal-directed practice in a smart environment).	Empowerment: AI operates as a resource/scaffold—instant feedback and visual progress records build personal resources (confidence and value), stimulating active engagement rather than passive reliance.
	21	Value–attitude beliefs	Intentionality	Belief basis: Motivation and engagement (value-driven behavioral engagement). Agency evidence: Agency (AI enhances willingness and ability for creative expression).	Empowerment: By scaffolding and personalizing experiences, AI reduces learning barriers and strengthens agency/creativity rather than replacing the creative process.

The included literature also indicated that cognitive dependence tended to present in four identifiable risk forms. First, the externalization of evaluative authority coupled with affective-interaction dilemmas: learners tended to cede the power to validate self-worth to algorithms and developed excessive expectations regarding AI scores, pass rates, or anthropomorphized feedback. Such reliance on external affirmation not only narrowed the source of confidence but, when AI exhibited strongly human-like features, could also trigger an uncanny valley effect, producing discomfort and psychological avoidance; this technology-induced affective barrier, in turn, could disrupt otherwise functional cognitive interaction (S9). Second, algorithmic disciplining and self-censorship: when creative processes were constrained by platform moderation and visibility rules, learners might proactively modify their creative inputs to fit algorithmic preference or ensure approval, resulting in algorithm-accommodating self-censorship and, consequently, a contraction of expressive intention and weakened agency (S2). Third, inertia in cognitive participation: some learners displayed productive deficits, in which they recognized a tool’s usefulness yet ignored its feedback; this behavior reflects a form of inert engagement in cognitive participation (S17). Fourth, deeper attributional bias and systemic anxiety: notably, superficial increases in confidence (i.e., higher self-efficacy) are not equivalent to deep internalization of cognitive structures. Evidence suggested that some learners reported increased confidence while their academic locus of control remained unchanged, continuing to attribute success to external factors (e.g., tool assistance) rather than personal effort—revealing a latent psychological dependence (S10). In addition, dependence was accompanied by anxiety about systemic risks, including concerns about algorithmic fairness and infrastructure stability, as well as fears that AI might ultimately replace human intelligence or deprive learners of interpersonal mentorship support (S12, S15).

### 5.4 Mechanisms of transition from scaffolding to a dependence trap

Based on the integrative synthesis of the included evidence, three progressively layered pathways were identified. The first operates at the micro-level of information processing: the extent to which AI feedback is embedded in an internalizable learning loop. This mechanism appears to function as a cognitive starting point that differentiates empowerment from dependence. When AI feedback is incorporated into a learning loop that involves understanding underlying causes, making revisions accordingly, and engaging in reflection and validation, it is more likely to function as cognitive scaffolding that supports internalization. Conversely, when feedback interaction remains at a surface level characterized by the provision of answers, the assignment of scores, and pass–fail determinations, learners are more likely to outsource evaluative judgment to the system, thereby increasing the risk of dependence. This pattern was corroborated across multiple studies. Findings in S1 suggested that “AI alone is not sufficient,” whereas when dialogic feedback and audio comparison were combined with reflective journals, external prompts were more likely to be transformed into internal self-monitoring and strategy optimization. Similarly, the relatively stable empowerment effects observed for the assessment-oriented app in S8 appeared attributable to embedding technological support across the full cycle of self-regulated learning—from goal setting to process monitoring and outcome reflection—thereby enabling AI feedback to become part of learners’ regulatory loop. Visualized feedback in S16 exhibited a comparable tendency: when seeing sound provided objective evidence for self-evaluation, learners showed a stronger willingness to bring the tool into extracurricular self-directed practice, thereby supporting a sustained process of self-diagnosis and subsequent correction.

The second pathway concerns task design—specifically, how systems reshape the challenge structure of learning tasks. Along this dimension, whether AI enables empowerment depends partly on whether it can maintain learning within a productive challenge zone through a mechanism of difficulty titration. On the one hand, positive empowerment often arises from the precise maintenance of challenge. When systems use adaptive calibration to keep tasks within a range that is “effortful yet achievable,” they are more likely to initiate a virtuous cycle in which learners become more willing to practice, are able to sustain practice over time, and can clearly perceive their own improvement (S3, S13). Available evidence suggests that this mechanism not only strengthens confidence and perceived career utility via visualized progress evidence (S18) but also, under the moderation of technology affinity, facilitates highly focused flow states (S20). Within this framework, S5 and S6 provide strong evidence that generative AI can function as a capability amplifier: successful empowerment does not necessarily remove difficulty; it may instead involve appropriate cognitive offloading. When systems such as DeepBach assume laborious computation and thereby shift learners toward higher-order aesthetic decision-making and knowledge application (S5), or when task decomposition supports the accumulation of successful experiences that enhance self-efficacy and emotional intelligence (S6), AI effectively reconstructs the dimensions of challenge rather than depriving learners of learning opportunities. On the other hand, imbalanced challenge structures may increase the likelihood of sliding into dependence. One risk concerns agency loss through oversimplification: S12 documented learners’ concerns that “AI may fully replace human intelligence,” implying that when generative tools offer one-click, template-like accompaniments without meaningful cognitive participation, learners may experience deeper anxiety about constrained skill development and loss of subjective value. A second risk concerns goal distortion leading to productive deficits. S17 observed that some learners engaged in repeated trials primarily to obtain high scores (e.g., up to 32 attempts to get a 10), while knowingly ignoring corrective feedback during practice. This phenomenon suggests that when AI scores become the dominant motive, the learning loop may be distorted into mechanical trial-and-error for a higher score, such that high performance conceals shallow self-regulatory strategies and the substantive guidance function of feedback becomes marginalized.

The third pathway derives from human–AI orchestration, namely whether AI is positioned as a substitute for relationships or as an enhancer, and what cognitive intention learners bring to the interaction. S18 showed that combining teacher guidance with students’ autonomous app use maximized psychological gains, implying that the most effective pathway is not AI-only but role-differentiated orchestration: AI provides objective evidence and immediate feedback, whereas teachers provide value guidance and affective support. However, departures from this configuration may engender deeper, environmentally induced dependence. The first concern is substitution risk and ethical deficits: when technology is designed to replace mentorship, or when infrastructure instability and algorithmic fairness concerns arise, learners may feel passively constrained and at risk of ceding agency (S15). The second concern is the potential backlash of anthropomorphic design: if over-humanized AI triggers the uncanny valley effect, learners’ attention may shift from skill acquisition toward seeking psychological certainty, and they may even become dependent on hybrid human–AI review to buffer affective discomfort (S9). A more covert risk lies in potential misalignment of psychological attribution. S10 provides an important warning: even when AI increases self-efficacy, the academic locus of control may not change. This implies that learners’ confidence may rest on tool support rather than personal capability, leaving a dependence vulnerability whereby functioning deteriorates once the tool is removed. Therefore, the key variable that ultimately determines pathway direction appears to return to learners’ cognitive agency and critical intention during interaction. S11 and S2 offer a striking contrast: when learners maintain independent aesthetic commitments and position AI as a tool for active questioning, hypothesis testing, and reflective support, the system is more likely to be domesticated into an intelligent partner that promotes knowledge restructuring and creativity (S11). By contrast, when learners lack internal evaluative criteria, they may readily treat AI as absolute authority or an aesthetic endpoint, falling into a passive stance of self-censorship aimed at accommodating algorithmic standards (guessing what the AI wants), thereby weakening agency (S2).

## 6 Discussion

**Figure 4:**
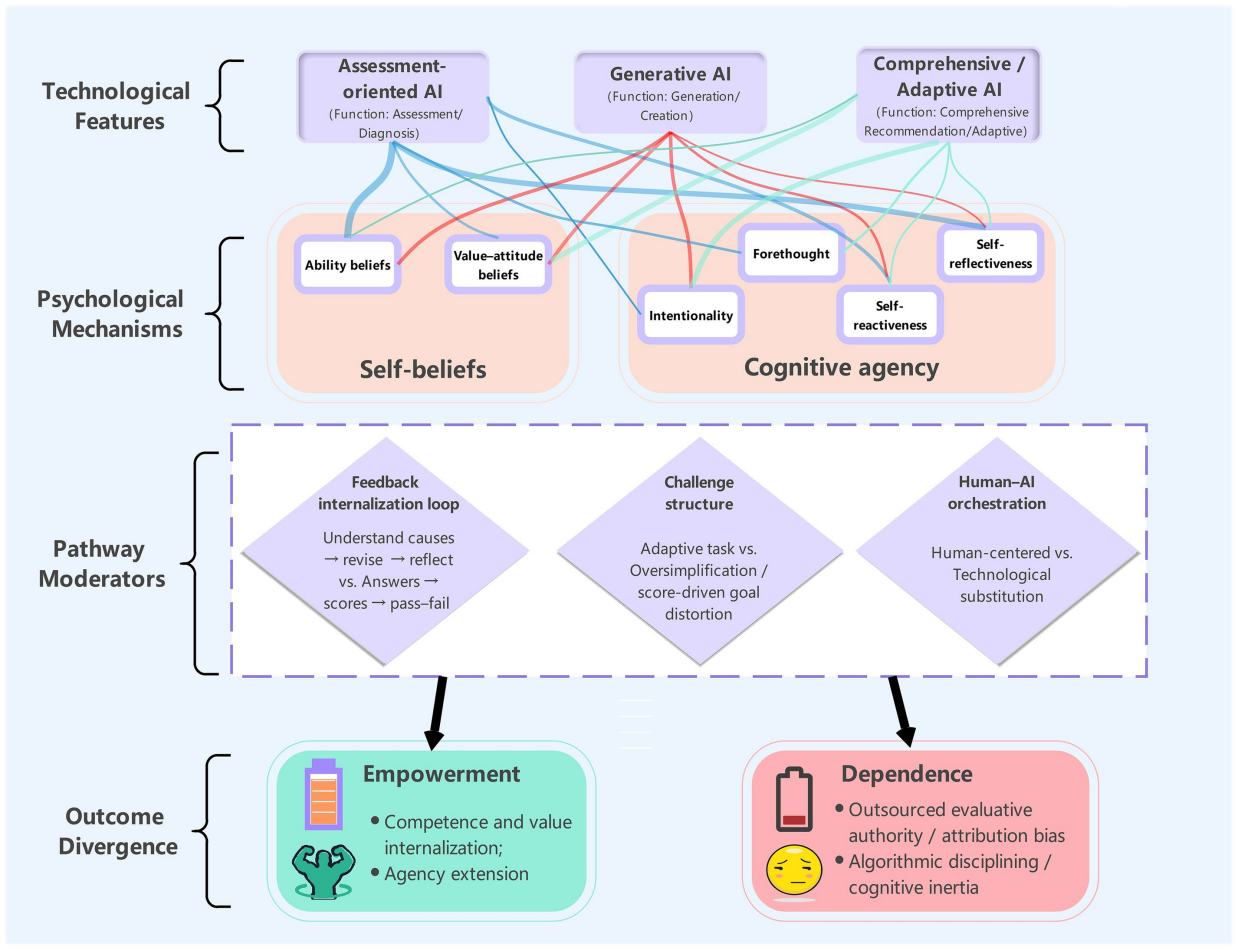
Conceptual framework: AI tool types, psychological mechanisms, and outcome divergence.

This review sought to move beyond a narrow verification of “technological effectiveness” and to elucidate the pedagogical and cognitive mechanisms that steer the direction of AI efficacy, with particular attention to how different technological pathways reconfigure learners’ self-beliefs and the boundaries of cognitive agency. Through a systematic synthesis of the extant literature, the present study first delineates an overall evidentiary landscape of the field: geographically, the available studies are highly concentrated in China and, more broadly, East Asia; developmentally, samples are markedly skewed toward higher education; methodologically, the evidence base is dominated by quantitative accumulation complemented by more limited qualitative mechanism-oriented interpretation. From the standpoint of sample scale, the field currently exhibits two coexisting traditions—thick-description, small-sample mechanism probing and large-scale correlational/model-testing approaches. Building on this evidence map, the review addresses the three research questions and articulates the deeper logic linking technological intervention to psychological reconfiguration (see Figure 4).

To begin with, regarding how different AI tool types shape psychological structures, the findings indicate that AI’s psychological effects are not homogeneous technology gains, but are strongly contingent on a tool’s functional positioning and interaction logic. Specifically, assessment -oriented AI tends to strengthen learners’ ability beliefs via quantified external evidence (e.g., pitch contours, rhythm scores) and to orient cognitive agency toward self-monitoring and benchmarked practice. By contrast, generative AI primarily reinforces value-attitude beliefs by lowering technical barriers and reconfiguring creative roles, while shifting cognitive agency toward higher-order intentionality, aesthetic decision-making, and knowledge restructuring. Comprehensive/adaptive AI further anchors psychological benefits in the dynamic maintenance of challenge–ability alignment, making it more likely that flow, forethought-based regulation, and active exploration can be sustained throughout learning processes. These conclusions extend the established tradition on technology-enhanced music learning (Lin and Chen, 2025; Dai, 2021). The key contribution of the present review is to show that AI tool types differ not merely in the magnitude of learning outcomes, but—more fundamentally—in the distinct psychological variables and cognitive pathways they activate.

In parallel, with respect to the concrete psychological manifestations, AI in music learning more often yields a coexistence of empowerment and dependence rather than a unidirectional effect. Psychological empowerment is typically expressed as gains in perceived competence and control, heightened learning motivation and engagement, and the externalization of self-regulated learning behaviors, aligning with prior observations (Wu et al., 2023). Cognitive dependence, however, more frequently takes the form of externalized evaluative authority, inflated expectations of algorithmic feedback with affective pull, and goal distortion and weakened agency under score-oriented or platform-rule-oriented contexts, corroborating earlier concerns (Fan et al., 2024). In other words, the central issue is not whether AI works, but through which mechanisms it works—and where those mechanisms may reverse. Notably, although primary- and secondary-school samples remain scarce, the present synthesis suggests developmental heterogeneity in the forms of dependence. In primary-school contexts, learners appear more prone to treat software scores as direct goals and to organize practice around point maximization (López-Calatayud and Tejada, 2024), implying that younger learners may construe AI as an external reward–punishment device or a scoring referee rather than a reflective tool. Evidence from secondary education points to a different sensitivity: amid heightened identity-construction demands in adolescence, generative AI and platform rules can jointly produce a being watched/being evaluated pressure, encouraging self-censorship or algorithm-accommodating strategies; creative setbacks may then more readily destabilize identity-related beliefs (Zhu, 2025). University learners, by comparison, more often demonstrate agentic strategic domestication of tools—for example, using peer verification and hybrid review to address algorithmic uncertainty (Tan et al., 2025). This developmental pattern implies that dependence is not invariably a passive decline; it may also include strategic attempts to introduce external constraints for social calibration.

Importantly, concerning the mechanisms that determine efficacy trajectories, the present review suggests that whether AI supports agency does not hinge primarily on feedback accuracy or speed, but on a reconfiguration of a threefold interaction logic. First, when AI feedback is embedded within an internalizable learning loop that involves understanding underlying causes, attempting revision, and engaging in reflective validation, it is more likely to operate as scaffolding that can be progressively faded; when interaction remains at the surface level of providing answers, assigning scores, and determining pass–fail outcomes, learners are more likely to outsource judgment to the system, increasing dependence risk. This pattern accords with the scaffolding principle that support should promote internalization rather than permanent substitution (Wood et al., 1976) and resonates with the cyclical monitoring emphasized in models of self-regulated learning (Zimmerman, 2000). Importantly, this mechanism has been explicitly articulated in research on computer-based scaffolding, which demonstrates that technological feedback promotes learning most effectively when it is designed to scaffold metacognitive monitoring and is progressively faded to encourage internal control (Azevedo and Hadwin, 2005). Relatedly, dialogic reflection highlighted by Li et al. (2025) and whole-cycle SRL support described by Ou et al. (2025) both underscore that translating external prompts into internal regulation is pivotal for empowerment. Second, at the level of challenge structure, empowerment more readily occurs when cognitive offloading is used to liberate space for higher-order thinking, rather than when over-simplification or score-driven goal distortion deprives learners of opportunities for skill acquisition. Evidence indicates that adaptive difficulty—dynamically adjusting task demands to learners’ moment-to-moment performance—can provide immediate scaffolding and improve learning performance relative to non-adaptive designs (Faber et al., 2024). Conversely, from the perspective of skill-learning theory, overly dense or overly strong external feedback may produce the dependence risks described by the guidance hypothesis: performance improves in the presence of feedback, yet retention and transfer are constrained once feedback is withdrawn (Salmoni et al., 1984). Third, at the level of agentic intention, a critical human–AI orchestration that remains human-led, technology-supported is necessary to prevent aesthetic intuition from being colonized by algorithmic disciplining. This necessity is echoed in the empirical findings of Zhang and Li (2025): although AI intervention can significantly improve quantifiable achievement, such technology gains do not automatically translate into affective connection and experiences of being cared for within pedagogical relationships. Accordingly, in music education—where sensory acuity, reciprocal interpersonal feedback, and value orientation are central—clear human–AI functional boundaries are warranted: AI should be positioned as a technical node for objective evidence, process scaffolding, and repetitive skill practice, whereas teachers and peer communities remain indispensable for aesthetic critique, meaning negotiation, and deep affective support. This human-centered logic aligns closely with UNESCO’s (2023) guiding report. Ultimately, the boundary between empowerment and dependence rests on the preservation of agency: when learners actively question, validate, and treat AI as one link in a broader toolchain, empowerment is more likely to emerge; when algorithmic outputs are treated as terminal authority, dependence becomes consolidated.

Beyond these core mechanisms, the review also notes Liu’s (2025) observation that female learners performed better when using AI-based affect visualization tools. Although most studies reported gender proportions, few systematically tested gender as a moderator, indicating the need for more fine-grained investigation of demographic variables in human–AI interaction.

Finally, while this systematic review identifies multiple pathways through which AI may shape music learners’ agency, several limitations warrant cautious interpretation. First, linguistic and cultural homogeneity may introduce bias. Because the search was restricted to English-language publications, the evidence base is geographically concentrated in China and East Asian educational settings. Given the cultural embeddedness of music education, East Asian traditions emphasizing respect for teachers and authority (Ahmed and Jielin, 2017; Huang and Asghar, 2016), together with highly competitive examination cultures (Chen, 2023), may predispose learners in these contexts to exhibit stronger compliance or technological dependence toward AI scoring systems as a novel authority substitute. Whether the dependence mechanisms synthesized here generalize to Western music-education contexts that emphasize individualism, critical thinking, and improvisational expression remains to be established through future cross-cultural comparative evidence. As a consequence, the dependence pathways identified in this review may be disproportionately shaped by authority- and examination-oriented learning ecologies, which reduces confidence in their transferability to contexts that prioritize improvisation, learner autonomy, and dialogic critique.

Second, sample distribution reveals a pronounced educational-stage discontinuity: the literature is heavily concentrated in universities, with relatively few studies in primary and secondary education and an evident absence in preschool contexts. This discontinuity not only limits a panoramic understanding of AI’s effects but may also conceal ethical and cognitive risks distinctive to basic education. In particular, the predominance of university samples may amplify the strategic use by mature learners (i.e., learners with stronger metacognitive capacity to manage AI). In contrast, the limited evidence in primary/secondary education suggests that younger learners more readily reframe AI as an external reward–punishment device or scoring referee. Due to sample constraints, however, it remains unclear whether this reflects a developmental age effect or a context-dependent consequence intensified by insufficient pedagogical guidance or correctness-oriented evaluation cultures prevalent in basic education. Future work should expand age coverage and use cross-stage comparative designs to disentangle net effects of developmental constraints versus contextual induction, thereby clarifying when and how younger learners can achieve agentic mastery of AI through appropriate orchestration. Therefore, any stage-related claims in the present synthesis should be treated as provisional, because observed differences may be confounded by contextual factors (e.g., assessment regimes, orchestration quality, and classroom norms) rather than reflecting developmental age effects per se, which weakens the reliability of age-based generalizations.

Third, limitations exist in evidentiary strength and causal inference. Although the review aims to construct a mechanism model, many included studies rely on cross-sectional designs or short-term interventions; rigorous experiments or mediation tests that tightly control the full causal chain—linking technological features to psychological variables and then to agentic behavior—remain insufficient. Crucially, these cross-sectional designs capture only immediate efficacy or short-term psychological fluctuations. They fail to reveal whether empowerment persists or if cognitive atrophy develops after tool withdrawal. Therefore, the observed positive outcomes may partially reflect a novelty effect rather than genuine internalization, thereby limiting the reliability of conclusions regarding the sustainability of cognitive agency. Consequently, some pathways identified here are derived primarily from theoretical coherence and cross-study pattern convergence rather than from tightly controlled longitudinal tracking or experimental manipulation within single studies. This may overestimate the generality of certain psychological mechanisms and makes it difficult to fully rule out confounding from individual differences (e.g., baseline motivation).

Fourth, the scarcity of long-term evidence is a shared limitation in the field. At present, there are insufficient longitudinal data to determine whether sustained reliance on AI support ultimately yields internalized gains or degradation in independent aesthetic judgment once the tool is removed. Addressing this gap requires a shift from short-term immediate efficacy validation toward longer-horizon examination of ecological literacy trajectories. This substantially constrains the reliability of any long-horizon conclusion about whether AI ultimately produces durable empowerment (internalized agency) or delayed dependence (erosion of independent judgment), because both trajectories remain plausible under the current evidence base. Importantly, the convergence of geographical bias (predominantly East Asian), educational stage gaps (mostly higher education), and methodological homogeneity (cross-sectional surveys) significantly narrows the current evidentiary scope. The apparent consensus may essentially reflect specific contextual artifacts rather than universal psychological mechanisms across diverse musical cultures and ages. Thus, this review represents a snapshot of an uneven, rapidly evolving field, highlighting an urgent need for diverse research designs to underpin more robust conclusions.

Taken together, these constraints imply that our conclusions are strongest for mapping recurrent tool–psychology associations across the current literature, whereas claims about cross-cultural generalizability, developmental stage effects, and long-term trajectories of empowerment versus dependence should be regarded as tentative and prioritized hypotheses for future longitudinal and comparative testing.

## 7 Future research

Drawing on the systematic heatmap analysis presented in Figure 4, the present review identifies a salient construct–tool mismatch in the field and proposes key avenues for future inquiry. Beyond addressing the construct–tool mismatch, future research must confront the field’s current contextual and methodological blind spots. Priority avenues include: (a) Cross-cultural verification to test whether dependence pathways observed in exam-oriented ecologies generalize to contexts emphasizing improvisation and dialogic critique; (b) Cross-stage designs spanning primary to university levels to disentangle developmental factors (age/competence) from environmental confounds (assessment cultures); and (c) Methodological diversification, integrating qualitative process tracking with longitudinal assessments—specifically evaluating post-withdrawal effects—to distinguish genuine internalized gains from latent delayed dependence. Given the rapid iteration of AI tools and their susceptibility to local ecological shaping, these diverse lines of inquiry are urgently needed.

First, an urgent priority is to address the near-complete absence of research on forethought within the cognitive agency construct in generative AI contexts. The heatmap indicates that, relative to other dimensions, forethought is sparsely discussed overall, and it is entirely absent in generative AI studies (*n* = 0). Two explanations are plausible. On the one hand, the end-to-end generation logic of generative AI may encourage cognitive offloading, prompting learners to bypass traditional phases of task analysis, goal setting, and strategic planning—effectively producing a technological delegation of forethought. On the other hand, definitional lag may obscure substantive forethought: iterative prompt optimization may itself constitute a novel form of forethought, yet has not been sufficiently theorized or empirically examined. Future empirical work should therefore focus on process-level analyses of human–AI co-planning, comparing cognitive investment during preparatory stages in traditional creation versus AI-assisted creation, and testing whether AI eliminates planning opportunities or instead requires forethought to be transformed into higher-order instruction design competence.

**Figure 5:**
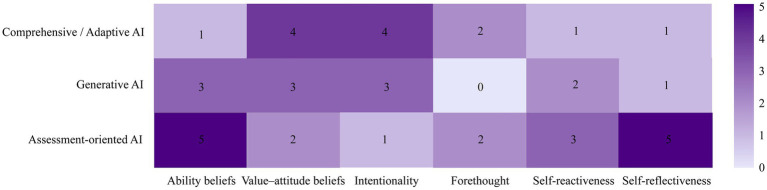
Construct–tool heatmap for evidence coverage across AI types and psychological dimensions.

Second, as Figure 5 shows, although comprehensive/adaptive AI may stimulate value-attitude beliefs and intentionality, evidence remains scarce regarding its capacity to elicit self-reactiveness (*n* = 1) and self-reflectiveness (*n* = 1) within cognitive agency. This gap suggests that current research emphasizes how algorithms adapt to learners while underexamining whether learners correspondingly abandon self-regulation. Longitudinal studies should explicitly test the algorithmic outsourcing risk by examining whether sustained reliance on adaptive recommendation weakens learners’ metacognitive capacity to autonomously monitor and regulate learning trajectories.

Third, the functional boundary of assessment-oriented AI should be expanded such that it evolves from a purely diagnostic tool into an intentionality engine. Although evidence is relatively robust for assessment-oriented AI in strengthening ability beliefs (*n* = 5) and self-reflectiveness (*n* = 5), its association with intentionality appears weak (*n* = 1). This pattern may indicate that current AI scoring primarily supports knowing one’s score (capability confirmation) and knowing what is wrong (reflection), yet does not consistently translate into knowing what I will do to improve (intention generation). Future research could experimentally compare different forms of AI data visualization (e.g., simple error curves vs. dynamic comparisons between ideal models and actual performances) to test how discrepancy representations shape learners’ psychological decisions. The central question is which form of gap presentation most effectively elicits a strong impulse to reduce this discrepancy (i.e., intentionality). In addition, instructional design research should go beyond more accurate scoring and instead explore how explanatory feedback can translate external evaluation into learners’ internal intention to engage in active revision, thereby enabling an agentic shift from being evaluated to actively seeking understanding.

## 8 Implications for instructional design and ethical practice

This study offers three key implications for instructional design and ethical practice in music education in the age of intelligent technologies. Educators in non-exam-oriented contexts or basic education settings should adapt these strategies to their specific ecological conditions, rather than applying them mechanically. First, at the level of instructional design, the integration of AI must shift from substituting human judgment to fostering internalizable learning loops. Rather than functioning as a surrogate decision-maker, AI should serve as cognitive scaffolding—providing explanatory feedback and guided reflection—that deliberately fades over time. This distinction is critical for primary and secondary learners, who are particularly susceptible to score-driven alienation where external metrics displace internal monitoring. To prevent short-term cognitive offloading from degenerating into long-term cognitive atrophy, and to operationalize these principles into concrete practice, we propose differentiated strategies categorized by tool type and educational stage.

Regarding Assessment-oriented AI, the core objective is to prevent reliance on external validation. For primary learners, educators should implement delayed feedback protocols and blind practice loops—requiring self-assessment before viewing AI scores—to mitigate dependence on immediate visual cues; teachers play a critical role here in transcoding algorithmic data into actionable goals. In secondary education, the focus shifts to internalization; strategies should combine explanatory feedback with peer discussion and brief self-regulated learning (SRL) logs to move attention from compliance with scores to understanding causes. At the university level, a fading scaffolding model is recommended, gradually reducing feedback frequency to enforce independent audiation and metacognitive calibration.

Concerning Generative AI, the pedagogical imperative is to preserve creative agency. For primary students, AI outputs must be framed as raw materials requiring selection and iterative refinement, strictly prohibiting one-click submissions. Secondary students benefit from forethought scaffolding—such as structural planning templates—and “constrained creation tasks” designed to counteract stylistic homogenization and maintain necessary cognitive friction. For university students, AI should be positioned as a hypothesis generator; curricula should mandate counterfactual revisions and rigorous documentation of human–AI contribution ratios to refine high-order aesthetic judgment.

Finally, for Comprehensive/Adaptive AI, the goal is to safeguard learner autonomy. Primary education should employ guided autonomy, providing multiple pathway options that require justification to avoid fully automated scheduling. Secondary instruction should introduce counter-recommendation tasks, requiring learners to critically evaluate and occasionally reject algorithmic suggestions. At the university level, the emphasis lies on epistemic sovereignty, encouraging students to compare system-generated pathways with self-constructed plans and to engage in ethical discourse regarding feedback authority.

Second, at the curricular and task-design level, educators should be cautious of tool-centered orientations that privilege scoring, efficiency, or generative speed, as these may inadvertently reconfigure learning goals according to algorithmic logics. Instead, instructional designs should deliberately preserve a degree of cognitive friction and aesthetic indeterminacy, thereby safeguarding the spaces of exploration, trial-and-error, and meaning-making that are intrinsic to musical learning.

Finally, at the level of educational ethics, it is essential to articulate a clear boundary of responsibility grounded in a “human-led, technology-supported” principle. AI should be positioned as a technical instrument that provides evidence-based support and cognitive extension, while value judgments, aesthetic guidance, and emotional care remain firmly anchored in teachers and learning communities. Only by safeguarding learners’ cognitive agency at both the design and ethical levels can AI realize genuine empowerment in music education, rather than evolving into a latent structure of dependence.
